# Preservice Elementary Teachers’ Socioscientific Reasoning During a Decision-Making Activity in the Context of COVID-19

**DOI:** 10.1007/s11191-022-00359-7

**Published:** 2022-07-01

**Authors:** Heesoo Ha, Wonyong Park, Jinwoong Song

**Affiliations:** 1 Center for Educational Research, Seoul National University, 1 Gwanak-ro, Seoul, 08826 Republic of Korea; 2 Southampton Education School, University of Southampton, University Road, Southampton, SO17 1BJ UK

## Abstract

The ongoing COVID-19 pandemic has highlighted the role of informed decision-making in times of crisis and the need for equipping teachers with the ability to address socioscientific issues in the classroom. In this study, we examine the features of socioscientific reasoning found in preservice elementary teachers’ group discussions on the issue of school reopening during the pandemic. Using socioscientific reasoning and perspective taking as theoretical lenses, we analyzed how the participants constructed and justified arguments about the issue from the perspectives of three stakeholders the Minister of Education, a teacher, and a parent. The analysis revealed the participants’ tendency to reach a premature decision and then cherry-pick evidence supporting the predetermined conclusion. As they examined relevant evidence, they often specified their initial claims by adding conditions to make it less objectionable and more defensible. We also illustrate how they used two different types of evidence, mechanistic and epidemiological, to support their claims about school reopening, and how perspective taking influenced their reasoning processes. Based on these findings, we discuss the potential of the perspective-based approach for supporting elementary teachers’ decision-making about socioscientific issues.

## Introduction

The COVID-19 pandemic has impacted education in myriad ways, from the immediate effects such as changes in teacher practices following the transition to online learning (Carrillo & Flores, 2020) to the long-term effects such as the decrease in teacher retention rates due to the economic uncertainty (Hannay et al., 2020). As the virus rapidly spread to the world, governments and international health authorities such as the World Health Organization were charged with two main tasks: first, to investigate the disease scientifically, and second, to manage and control the crisis by various social and political measures. One characteristic of science that stands out in this time of crisis is that we do not have empirical knowledge base that is sufficient for making decisions about the actions to be taken. It needed some time from the start of the outbreak for scientists to be able to produce any evidence (e.g., the effectiveness of face covering, social distancing, and antiviral medications) that can inform policy decisions. Science-in-the-making in times of crisis is distinct from the image of science as we normally think of it (Funtowicz & Ravetz, 1993). As Funtowicz and Ravetz (1993) contended, the “normal” mode of science is not very helpful in understanding solving complex social problems when facts are uncertain, values in dispute, and stakes high, as in the example of the current pandemic.

Scientifically informed decision-making is required not only for policymakers but also for diverse stakeholders in society, particularly when there exist conflicting interests and values about the decision to be made. As responsible citizens, individuals are expected to make important decisions as stakeholders of various issues in their lives such as school openings; Toleration of diverse viewpoints is a core value that drives democracy (Bohman, 2006). Every member of society, not only those making policies, is responsible for making scientifically informed decisions about pressing societal issues such as a pandemic (Pietrocola et al., 2021). A recent report by the Royal Society suggest that a scientifically informed individual “makes informed interpretations and judgements (e.g., risk assessment) about scientific information and the world at large as well as engaging constructively in debate on scientific issues” (The Royal Society Science Policy Centre, 2014, p. 29). Given the significance of science-based decision-making about COVID-19, the role of teachers in providing young citizens with the opportunity to develop such skills becomes critical. Research suggests that teachers play a vital role in how social issues are understood by students (Lazarowitz & Bloch, 2005; Lee et al., 2006). In this study, we specifically focus on preservice elementary teachers’ (PETs) engagement with socioscientific reasoning. It is important to focus on PETs for several reasons. First, researchers have highlighted the need for engaging in social issues from early years of education (Bautista et al., 2018). Second, elementary teachers are typically responsible for a range of subjects, which can allow for more room for socioscientific issues (SSI) that are inherently interdisciplinary and relevant for students (Broggy et al., 2017). Third, despite its significance, the incorporation of socioscientific issues into elementary schools is still rare relative to that into secondary schools (Kinskey & Zeidler, 2021).

This study utilized a decision-making activity on COVID-19 that incorporates different perspectives about an issue in a preservice elementary teacher education context. We draw on previous studies on decision-making on SSIs, or “ill-structured problems that involve moral, ethical, and financial aspects, and lack of clear-cut solutions, … that emerge at the nexus of science and society and have a degree of uncertainty” (Evagorou & Dillon, 2020, p. 2) and often involve “complex, open-ended, [and] often contentious dilemmas” (Sadler, 2004, p. 514). Studies on SSI have investigated students’ reasoning with a range of foci such as the role of scientific knowledge (Lewis & Leach, 2006; Nielsen, 2012), the use of evidence and reasoning from multiple perspectives (e.g., Evagorou et al., 2012; Kolstø, 2006; Lee & Grace, 2010, 2012), and the intertwinement of reasoning and values (e.g., Albe, 2008; Kolstø, 2006; Levinson, 2006; Nielsen, 2012; Rundgren et al., 2016).

Empirical studies that examined elementary school students’ socioscientific reasoning have suggested the necessity of teachers’ instructional support for constructing informed and reasoned decisions (Evagorou, 2011; Ozden, 2020). Acknowledging that teachers’ understandings and beliefs about teaching SSI can be critical factors that determine whether and how they address SSIs in their classrooms (Lee & Witz, 2009), researchers have implemented SSI decision-making activities in the teacher education context to investigate how preservice elementary teachers design SSI lesson plans (e.g., Borgerding & Dagistan, 2018; Ladachart & Ladachart, 2021; Lee et al., 2012; Topcu et al., 2010). Although addressing SSIs in elementary school is central to cultivating responsible citizens from an early age (Evagorou & Mauriz, 2017; Ozden, 2020), evidence suggests that SSI instruction is often challenging for teachers, most notably due to the lack of subject matter knowledge and training required for teaching SSIs (Kinskey & Zeidler, 2021). These studies indicate the need to understand preservice teachers’ practices of developing socioscientific reasoning and search for instructional strategies to support preservice elementary teachers’ knowledge of socioscientific reasoning.

The current study focuses on PETs’ decision-making in the context of COVID-19, particularly school reopening. A decision-making activity was designed and implemented to facilitate PETs’ consideration of multiple stakeholders and the use of relevant scientific information. The study was guided by three research questions as follows:

1. How are claims about SSIs generated and revised by of PETs?
2. What are the types of evidence that PETs use in their socioscientific decision-making?
3. How do PETs interpret and use the same piece of evidence in different ways to support perspectival claims?

## Theoretical Background

### Informal Reasoning in the Context of Socioscientific Decision-Making 

While formal reasoning is characterized by reasoning with fixed premises, informal reasoning is based on unstable and less accessible information regarding open-ended and ill-structured problems (Means & Voss, 1996). As Zohar and Nemet (2002) note, informal reasoning “involves reasoning about causes and consequences, advantages and disadvantages, or pros and cons of particular propositions or decision alternatives” and “underlies attitudes and opinions, involves ill-structured problems that have no definite solution, and often involves inductive (rather than deductive) reasoning problems” (p. 38). In the context of COVID-19, using statistical analysis to predict the number of infections in the future is an example of formal reasoning; making policy decisions based on such a prediction would be an instance of informal reasoning. Given the open-ended and controversial nature of SSI, decision-making about SSIs tends to have characteristics of informal rather than formal reasoning (Sadler, 2004; Sadler & Zeidler, 2005; Zohar & Nemet, 2002).

Sadler et al. (2007) conceptualized informal reasoning about SSI as “socioscientific reasoning” and described it as “a theoretical construct which subsumes aspects of practice associated with negotiation of SSI and addresses the citizenship goal” (p. 374). Specifically, socioscientific reasoning can be characterized as informal reasoning used to justify claims about SSIs (e.g., what the cause of the issue is, who is responsible for the issue, how it could be resolved). According to Sadler et al. (2007), the key aspects of socioscientific reasoning include “recognizing the inherent complexity of SSI, examining issues from multiple perspectives, appreciating that SSIs are subject to ongoing inquiries, and exhibiting skepticism when presented potentially biased information” (p. 374). Among these aspects, in this study, we focus on the value of multiple perspectives and explore how different perspectives are considered as PETs engage in informal reasoning on SSIs.

Although often loosely structured than formal reasoning, socioscientific reasoning still include core elements of reasoning such as the generation and evaluation of arguments (Sadler, 2004; Zohar & Nemet, 2002). Evagorou (2011) examined elementary students’ practices in an SSI-based decision-making activity and found that while students could construct evidence-based decisions, the level of justification was limited. In another study by Ozden (2020) on elementary school students’ socioscientific reasoning, intuitive reasoning was found to be more frequently used than logical reasoning. These studies suggest that elementary students’ socioscientific reasoning skills are limited and thus highlight the need for supporting teachers who can help students develop such skills.

The first step to supporting teachers would be providing teachers with opportunities to engage in making informed evidence-based decisions. For this purpose, several studies have investigated socioscientific reasoning and decision-making in preservice teacher education (Ceyhan et al., in press; Ozturk & Yilmaz-Tuzun, 2017; Topcu et al., 2010). Topcu et al. (2010) is one of the initial studies that analyzed preservice teachers’ informal reasoning. They examined the quality of Turkish preservice teachers’ informal reasoning and showed that most of the participants’ reasoning was underdeveloped and did not consider perspectives other than their own. Ozturk and Yilmaz-Tuzun (2017) analyzed PETs’ socioscientific reasoning by their identifying decision-making modes (i.e., whether the reasoning is intuitive or evidence-based) and reasoning modes (i.e., what aspects of an issue were considered). Collectively, these studies show that, while the PETs have some capacity to generate evidence-based arguments, more opportunities are needed to enhance their ability to provide quality evidence and reasoning to support claims.

### Multiple Stakeholders and Perspective Taking in Socioscientific Reasoning 

In the socioscientific reasoning process, we are often faced with competing values and interests held by multiple stakeholders from different sectors of society (Solomon & Abelson, 2012). By stakeholders, we mean individuals, groups, or organizations that are concerned with, or have interests in a specific issue (Oxford English Dictionary, n.d.). SSIs often involve human activities, conflicts, and dilemmas, and do not have a single answer, which implies that there can exist multiple stakeholders with varying interests and conflicting arguments (e.g., Rehr et al., 2012; Warner, 2006). Examples of such issues in the context of COVID-19 can range from mandating mask-wearing in the classroom to making alternative high-stakes examination plans. On these issues, policymakers, principals, teachers, students, and parents have varying interests and arguments that often conflict with one another.

Kahn and Zeidler (2019) highlighted the importance of perspectives, or “how one perceives and interprets an issue,” in socioscientific reasoning, arguing that perspective taking is instrumental to activating empathy, resolving controversies, and constructing shared knowledge. Several studies have incorporated perspective taking into student-led activities by asking students to consider different perspectives regarding SSIs in students’ decision-making (Lee & Grace, 2010, 2012; Rundgren et al., 2016). Kahn and Zeidler (2019) discussed the complexity of perspective taking on SSIs by highlighting how rationalistic, emotive, and intuitive patterns of reasoning manifest in the perspective taking. These studies suggest that activities asking students to take multiple perspectives and consider conflicting interests of stakeholders can have the potential to support learners’ engagement in everyday decision-making and facilitate preservice teachers to be better equipped to implement SSI-related decision-making activities in the classroom. Based on this line of ideas, the current study aims to integrate varying interests and perspectives into informal reasoning about an SSI. It will shed light on perspective taking as a crucial component of socioscientific reasoning and contribute to the understanding of approaches to design SSI-related decision-making activities in the context of preservice teacher education.

## Methods

### Educational Context and Participants

The context for this study was an undergraduate biology course for preservice elementary teachers in a university in South Korea. Due to the COVID-19 restrictions, the course was delivered online, using technologies such as Zoom and Slack for lectures and group discussions. Because of this online learning environment, the PETs were encouraged to engage in group discussions whenever they can without time limit. The course was taught by the first author, and among 28 course attendees, 20 PETs voluntarily participated in this study. For the decision-making activity, the PETs were asked to form six groups, each consisting of four or five members. Each group was provided with a virtual space in Slack for text-based discussion, and they were encouraged to use Zoom if preferred. The length of each group discussion ranged from one to five hours.

The main goal of the decision-making activity was to enable the PETs to learn how to engage in the decision-making of SSI related to COVID-19 and learn that different perspective taking can result in various decisions on SSI. We expected the PETs to achieve these learning goals by engaging in the decision-making of SSI related to COVID-19 and reflecting on the process. The decision-making activity was designed based on the design framework for SSI instruction suggested by Sadler (2011), who suggested SSIs to be situated within an instructional sequence and to scaffold the PETs to connect relevant content knowledge. Based on this design principle, to support the PETs to confront scientific ideas and theories related to the issue, the activity was situated within a session about pathogens and human immunology. The concepts covered in this lecture included the types and transmissions of pathogens and the structure and mechanism of the human immune system. The expectation was that the lecture would provide the PETs with some background knowledge necessary for understanding the virus and the current status of the COVID-19 pandemic. This instructor-centered introduction to the relevant concepts was followed by an assignment asking to watch a 20-min video clip about coronaviruses on YouTube and answer questions about characteristics of the coronavirus and the spread of the virus as explained in the video. This pre-session assignment was arranged based on a design suggestion from Sadler (2011)—using media to support PETs to understand the scientific background of the issue and connect the conceptual learning to the real-world problem. The information from the lecture and the video clip provided background knowledge for the PETs to comprehend information they found during the decision-making activity, such as characteristics of COVID-19, development of treatments and vaccines to COVID-19 virus, and precedent pandemic cases by coronavirus.

In the decision-making activity that followed, the groups were asked to construct an argument about when to reopen elementary schools during COVID-19. This issue was chosen on the grounds that the decision-making on this issue would involve stakeholders from the perspectives that PETs could hold in the future. In addition, this issue was being actively discussed in Korean society at the time of the study. Thus, we expected that the PETs can take on the perspectives and reason on the issue. For a focused activity and discussion, we set the time point for decision-making to be the middle of April 2020 and asked the PETs to use the scientific information and disease statistics available by that time. To facilitate the PETs to approach the issue in different perspectives, the PET groups were assigned one of the following three perspectives: (a) The Minister of Education, responsible for both the operation of the national education system and the decision to reopen schools; (b) An elementary school teacher who is a third-grade homeroom teacher and in charge of student safety if the school reopens. We hypothesized that the teacher also teaches science and prefers enacting various activities and laboratory work in science lessons; (c) A parent with a child in third grade, in a dual-income family, and therefore regularly using childcare services. Two groups were assigned for each perspective. By engaging the PETs in the decision-making activity from these three perspectives, we aimed to promote the PETs’ empathy, which is an essential component of perspective taking and socioscientific reasoning (Kahn & Zeidler, 2019).

**Fig. 1:**
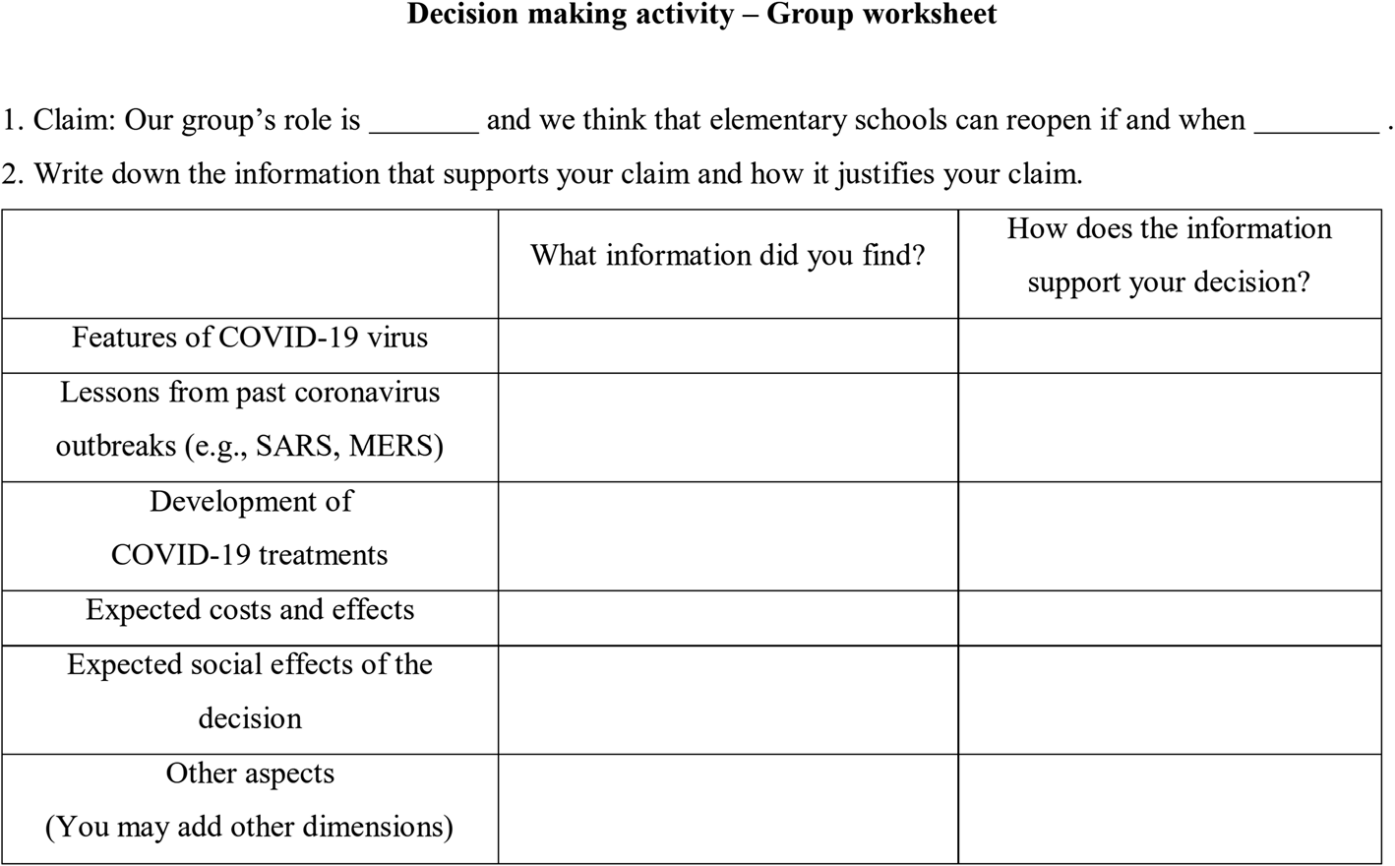
Group worksheet used for the decision-making activity

The PETs were asked to record their arguments in a worksheet (Fig. 1). Here, we use “arguments” to refer to the product of “argumentation,” the justification of claims based on evidence and reasoning (Erduran & Jimenez-Aleixandre, 2007). Therefore, the arguments produced by the PETs consisted of a “claim” about reopening schools, “evidence” they used to support the claim, and “reasoning” through which they related the claim and the evidence. To scaffold PETs’ construction of evidence-based decisions, the worksheet was designed with separate sections to write down the claim (the first item) and justification (the second item). In the justification section, the PETs were asked to collect information from various accessible sources to construct and justify their claims. To support the PETs to consider various aspects of the issue, we provided a table including five key dimensions of the pandemic that were potentially relevant to school reopening. The five key dimensions in the table were developed based on the epistemic tool of Ke et al. (2020) to support the PETs to “investigate the issue … and develop understandings of multiple aspects of the issue” (p. 7). Ke et al. (2020) developed a chart that provides spaces to write down information related to an SSI in multiple aspects such as science, ethics, and economics. We adapted the original tool by dividing each dimension into the “data” column (“What information did you find?”) and the “warrant” column (“How does the information support your decision?”) to explicate the PETs’ reasoning processes. Under the table, we provided hyperlinks to several useful sources regarding COVID-19 such as news articles and government announcements. The sources included a web page providing the statistical description of COVID-19 cases in Korea in the middle of April 2020, news articles about positive and negative forecasts on the development of COVID-19 and announcements of the government’s and schools’ COVID measures in April 2020. In selecting these sources, we focused on supporting the PETs to understand how people in different perspectives interpret the situation and respond in various ways. To support the PETs to construct justified decisions, the instructor encouraged them to refine decisions as they develop justification and provided sufficient time to complete the activity.

### Data Collection

The main data source was the PETs’ group discussion in Zoom and Slack. If a group chose to use Zoom video chat for group discussion, they were asked to record their discussion and submit the recorded video files, which were transcribed verbatim for analysis. The worksheets that the PETs submitted were also used for the analysis. These data sources provided information about the claims and evidence that were suggested, adopted or discarded in each group during the decision-making.

After the activity, each PET completed a survey to reflect on their engagement in the activity. The PETs’ answers were used as supplementary data to understand to what extent they felt that their perspectives influenced their decision-making processes. The survey questions were “(a) Did your group’s role affect your decision-making process? If it did, please explain how; and (b) What were the factors that you think all groups considered to be important in decision making? What were the factors that your group considered more important than the other groups because of your group’s role?” Responses to these reflective questions were used to nuance and contextualize the findings from the worksheets and group discussion recordings. For example, the survey responses contributed to understanding the rationales and intentions for making their decisions and capturing any disagreements within the group. The plans for data collection had been reviewed and approved by the institutional review board at Seoul National University for any potential ethical issues before the semester began. Informed consent was obtained from all participants.

### Data Analysis

The analysis of qualitative data was conducted in two phases. The first phase involved repeatedly reading the transcripts, survey responses and student worksheets to gain familiarity with data. We identified claim, data, and reasoning (Kolstø, 2006) in each group’s decision-making process, and summarized the flow of these identified components. To better capture how different perspectives were considered, we divided the “data” category data into two subcategories: (a) “evidence,” which includes information and knowledge relevant for making decisions, and (b) “interests,” which refers to the values and considerations arising from the stakeholders’ positions and responsibilities. Based on this analysis, the structure and components of each group’s decision were organized into a table (Table 1). Two of the authors individually wrote analytical memos in the transcripts (Birks et al., 2008) about the differences that we noticed in the discussions and decisions made by the PET groups. Then, we met and shared these notes, to reach a consensus on the analysis.

**Table 1:** An overview of decisions developed in each PET group

		Ministry of Education group	Teacher group	Parent group
Claim		We think that elementary schools can reopen (1) on the first Monday of September; (2) when the number of daily infection cases in Korea is under five (excluding people from overseas)—10% of 50 that is the criteria for social distancing level one as suggested by the Korean government; and (3) when there are less than two community infections	We think that elementary schools can reopen when the number of self-isolating people reaches zero and overseas acquired cases are completely under control	We think that elementary schools can reopen in the fall semester if the number of daily infected cases is less than 10 for at least two weeks
Stakeholder interests considered		Student safety, keeping the education system going	Student safety, teachers’ responsibility for student safety	Student safety, household economy
Evidence	Scientific	An expectation that the COVID-19 crisis would not end until July (news article) Possibility of re-spread of the virus in fall (news article)	An expectation that it will take too much time to end COVID-19 worldwide (news article)	The prospect of available treatments by the second half of the year (news article) Successful cases of plasma treatment (news article)
	Epidemiological	The number of daily cases when high schools partially reopened (news article) The number of daily cases when a mass virus infection occurred at clubs (news article)		
	Policy	The government standards for lowering the level of social distancing (news article)		Universities’ policies on reopening during COVID-19 (university announcements)

In the second phase, we selected one group from each of the three perspectives (i.e., the Minister of Education, a teacher, and a parent) that were information-rich (Yin, 2016) in light of the research question for an in-depth, comparative analysis. Then, we revisited the data, research memos, and the tables to characterize the features of the claims and the evidence used in each group. We also examined the similarities and differences among arguments made by the three groups, specifically as to how claims in different perspectives differ, which evidence is used to support which claim, how information about COVID-19 is interpreted differently, and what interests of stakeholders are considered. The trustworthiness of the analysis was achieved by comparing multiple data sources such as transcripts, student worksheets, and their responses to survey questions (Miles & Huberman, 1994). Two of the authors individually coded the data first and then went through discussions to reach a consensus and checked for possible rival explanations or any conflicting evidence (Yin, 2016).

## Findings

### Overview of the Socioscientific Decision-Making Processes

Table 1 presents an overview of the decisions made by the three focal groups. All groups, regardless of the specific perspective taken, commonly considered student safety as a major stakeholder interest. However, other distinctive interests that were emphasized in each perspective led the groups to reach different decisions about school reopening. For example, due to the responsibility to maintain the national educational system, PETs in *the Minister of Education group* considered that schools should reopen *before* the virus transmission ends and suggested several specific conditions for school reopening based on several pieces of evidence. In *the teacher group*, the focus on student safety was mainly associated with teachers’ responsibility for ensuring student safety in the classroom. Unlike the other groups who made decisions based on the government’s social distancing levels, the teacher group raised suspicions on the government policy and claimed that schools can reopen when the chances of transmissions in schools are low enough. *The parent group* used as the main evidence a news article about the high chance of treatments being developed in the second half of the year, claiming that schools can reopen in the fall semester if the number of daily infected cases can be kept low. This claim reflected the main concern of the group, which was to ensure student safety and household economy simultaneously. In the following, we further explain four main features of decisions and socioscientific reasoning identified across the three groups.

### Hasty Conclusions and Evidence Cherry-Picking

One predominant trend across the three groups’ reasoning processes was that all of them arrived at their decision about school reopening at an early stage of the group discussion. Such premature decisions tended to rely on their inferences about what the stakeholder would consider in the situation. The excerpt below at the start of the parent group’s discussion illustrates how their decision to stand against school reopening was established early in the discussion:

1 Yoon:… Personally, I would want to send my kids back to school.

2 Jean: But based on the given scenario, it sounds like the teacher wants the school to reopen.

3-9 Hyun: But childcare service kept running when schools did not reopen. … If classes are run online and only childcare service is run on-site in the schools, there would be just kids who need the service in schools. But if the school reopens, the entire students are going to gather. So, I will go against the school reopening if I am a parent.

26-29 Jean: On the side of the Minister of Education, the most important problem is that the education system is delayed. … The teacher will definitely stand against school reopening.

31 Yoon: Because they are responsible [for infection at school].

36 Noah: Let’s go against the school reopening.

37 Jean: Yeah. Let’s go against it.

These exchanges show that, although different views on whether to reopen schools were suggested by the group members, these views were not carefully deliberated before reaching a premature decision that “Let’s go against it [the immediate school reopening]” (Line 37).^1^ It is worth noting that in this conversation, there is no reference to empirical or statistical evidence related to health risks of COVID-19 to reach their premature conclusion.

Once a decision was reached, the subsequent conversations tended to be focused on selecting the evidence that can justify their decision. An example of such a “backward” process, where the conclusion appeared before the evaluation of evidence and the construction of reasoning, was observed in the Minister of Education group’s case. In the exchange below, it is explicit that the PETs were looking for evidence that will provide post hoc justification of their premature conclusion:

70 Yerin: We can make a claim like “Schools can reopen from the fall semester with a specific date, but if the second pandemic occurs, schools can reopen in the condition of …”

71–73 (The other PETs agree)

74 Hoon: Then we can emphasize the characteristics of the virus in relation to the temperature to justify the part about reopening from the fall semester.

75 Yerin: Let’s find out when elementary schools are scheduled to open in the fall semester at the moment.

76, 78 Hoon: Okay. … If we are going to claim that the school reopening needs to be postponed, we can talk about the [dangers of the possible] second pandemic.

Here, the group’s decision to reopen schools from the fall semester is already established at the beginning, and then the group members discuss what sorts of data would be needed to support their claim. In Line 74, Hoon relies upon the association between temperature and infection rate to support their reopening decision, which was not a verified scientific fact but was only suggested as a possibility in a news article they were looking at. Some alternative predictions such as low possibilities of the COVID-19 crisis to end in the year were also mentioned in the reference links in the worksheet that the PETs were given. These alternatives, however, were not considered by the group, and they reached a consensus only based on the evidence that was in favor of their decision.

This slanted selection of evidence and inattention to possible alternatives was also observed in the teacher group’s discussion. In this group, a possible alternative claim and reasoning from another perspective were raised during the discussion, but this concern was not deliberated in developing their reasoning:

115 Chan: Teachers are responsible for student safety in classrooms, so, it could have been different from the Minister of Education, but as for teachers, I think it’s reasonable to prefer [to reopen schools only after] the eradication of COVID-19.

116 Jeong: I agree. Teachers take the consequences if things go wrong. No one will share the burden.

117 Chan: That’s the reality.

118 Sue: Controlling interactions among students is also difficult. They are even supposed to have meals while socially distancing, but is it possible? That’s something to think about.

In this conversation, Chan raises a possible rebuttal to the popular idea that schools should reopen only when student safety is ensured, by referring to the attainment gap arising from the lack of teacher guidance in the online education environment (Line 115). Sue disagrees by referring to the difficulties in guiding students to keep their distance from one another in schools (Line 118). Despite the explication of these two opposing perspectives, the discussion ended as the PETs agreed that teachers were supposed to argue for reopening after a complete end of the virus transmission.

After reaching the decision based on teachers’ responsibilities, the group members looked for evidence that they individually found to support the agreed claim. Evidence that they mainly drew on was related to student safety. Potential problems with online education like the intensification of learning loss were also addressed, but they explained that “these problems should and could be overcome by providing high-quality online education.” Compared with the decisions developed by other groups, the value of online education as a substitute for face-to-face education was highlighted in the group’s decision-making as a result of their slanted selection of evidence and interpretation of data.

### Making Claims Weaker and Less Objectionable by Attaching Conditions

Once the groups reached a hasty conclusion, they tended to modify it by attaching conditions, in anticipation of possible rebuttals. This practice was particularly evident in the Minister of Education group. This group’s discussion on the conditions for school reopening was initiated by Yerin, who pointed out that “Because our role is the Minister of Education, we cannot *conclude* that ‘schools will reopen next year!’” (Lines 155–160, emphasis added). It was also described that they thought that one of their duties is “to consider students’, parents’, and teachers’ perspectives” (Hoon survey response). Although they were making decisions from a particular perspective, they were still conscious of the interests of other stakeholders. This suggests that the Ministry of Education group was concerned about their original claim being too strong and thus vulnerable to objections. As such, additional conditions were attached to the original claim to modify their claim to be acceptable to other stakeholders.

They attached conditions to their original claim that schools should reopen at a specific time. Examples of such conditions included “in the fall semester” or “next year.” Moreover, they also used more specific conditions pertaining to the COVID situation in the country. For instance, after Hoon’s suggestion for modifying their claim, Taek proposed adding the condition of reopening the schools “when the daily cases of infection in Korea falls below a certain number” (Line 161). It was also suggested that this number should be determined based on the government’s description of social distancing level, thus partially transferring the responsibilities for their decision to the authorities. As such, the groups attached conditions to their initial claim based on the government’s latest social distancing guidelines and made the claim more defensible.

Similarly, when concerns of practicality were raised about the suggested claim, the groups often modified their claim to make it “safer.” The conversation of the Minister group below indicates how their early claim to reopen schools after the country has been cleared of COVID-19 has evolved by addressing the possible rebuttal about the impact of school closure on student learning:

1 Yerin: Since our role is the Minister of Education, I think we cannot claim for closing schools until the country has been cleared of COVID-19.

2–6 Hoon: Wouldn’t it be enough to just say something about when schools will reopen and how teaching will happen until reopening? … I think we can continue online classes a little bit longer if guidelines can be provided.

28–30 Hoon: We can’t allow giving up a whole academic year since developing social skills is a goal of elementary education.

31 Min: It’s hard to decide.

32 Hoon: We can just say schools can reopen in the fall semester and focus on thinking about the guidelines and supports for face-to-face teaching?

In Line 1, Yerin points to the responsibility of the Minister of Education to argue against the idea of closing schools until the end of the pandemic. Hoon then proposes to revise the claim from a declarative to a conditional sentence, to address the concern raised by Yerin (Line 32). What this exchange shows is how they softened their claim by including measures to mitigate the impact on student learning. This strategy allowed them to make their argument more acceptable, without having to change the core of their initial claim about ensuring student safety.

### Use of Mechanistic and Epidemiological Evidence 

In the three groups’ decision-making process, two distinct types of scientific information were used as evidence: mechanistic and epidemiological evidence. Mechanistic evidence involves complex systems of multiple interacting components (Fagan, 2012) such as the molecular behavior of the coronavirus and its features that influence such behavior. On the contrary, we use the term “epidemiological evidence” to refer to information that pertains to the rates of infection in the population and usually come along with discussions of how public health measures can influence the spread of the virus. The PETs relied on both types of evidence, but there was a significant difference in the ways each type of evidence was linked to the decision about school reopening.

*Mechanistic evidence* was most frequently used to support the contagiousness of the coronavirus and to argue that student safety would be at risk thus school reopening should be postponed. For instance, the teacher group that claimed for reopening schools when there is no danger of infections throughout the nation wrote under the “Features of COVID-19” section in the worksheet that the coronavirus’s high fatality and contagiousness justify that “rash school reopening can cause another massive spread of the virus” and that “thorough preventive measures against infections in schools are needed.” Other groups used mechanistic evidence in largely similar manners, for instance, by detailing the molecular mechanism of the spread of the coronavirus through the air. This shows that the use of mechanistic evidence was fairly limited in the PETs’ socioscientific reasoning.

*Epidemiological evidence* was used, on the other hand, often combined with reference to the government’s social distancing levels, to contrive concrete timing and conditions for school reopening. Thus, except for the teacher group that claimed for postponing school reopening until there is no danger of infections, epidemiological information was the type of scientific evidence that was used to rationalize the decisions. One representative episode that illustrates this was found in the Minister of Education group. The discussion began with the PETs agreeing on postponing school reopening to the fall semester based on the expectation that the COVID-19 would not end until July. They realized that their thoughts on the timing for school reopening are not specific enough, and one PET suggested that “we need to discuss with what number of confirmed cases schools can reopen.” With this suggestion, the PETs searched for news articles about group infections along with the descriptions of the government’s social distancing levels:

178 Taek: So specifically what number of daily cases do we set as a criterion?

181 Yerin: What was the number of daily cases when the government’s social distancing level decreased last time?

185 (Min shares a news article reporting that there were 39 new cases that occurred on the day when the government’s social distancing level was lowered.)

191-202 Ran: So, I guess schools can reopen with fewer concerns when the daily cases are under that number. … Wait, did the group infections at clubs occur around that time?

207 Taek: The club case occurred on May 7^th^ (after the social distancing level was lowered).

231 Taek: Considering the club case, I think our criterion should have a number smaller than the government’s social distancing level on that day (when the club case occurred).

In this exchange, the PETs look for the number of daily confirmed cases at the time of a large number of infections in the country and suggest using that number as a criterion for deciding the condition for school reopening (Lines 178–185). They then evaluate the effectiveness of the government’s decision for social distancing level at the date when group infections occurred—whether they can follow the government’s decision as a condition for school reopening and decide how to modify the government’s decision to propose conditions for school reopening (Lines 191–231). This discussion was later incorporated into their final claim where they advocated stricter conditions for school reopening than the government’s social distancing level and included the number of community infections as one of the conditions (Table 1). This instance suggests that epidemiological evidence about a rapid increase of the rate of infection in a certain condition was used to evaluate whether a certain social distancing level is reliable enough to base their decision on.

### Perspectival Evaluation and Use of Evidence

Given the nature of perspective taking as the ability to differentiate others’ views from one’s own and systematically evaluate how those data fit within the realm of one’s cognitive and affective experiences (Kahn & Zeidler, 2017), the same piece of evidence can be interpreted and utilized differently. In our data, the comparison between the Education Minister group and the teacher group illustrated this point, particularly with regard to how the two groups used the Korean government’s three-tier social distancing system differently as evidence. In the Minister of Education group, the government’s social distancing levels were referred to in order to determine the conditions for school reopening. One group member referred to a news article about the government’s decision to lower the level, based on the significant decrease in the number of daily confirmed cases down to around 20.

On the contrary, the teacher group did not take the government’s social distancing level for granted in their decision-making. For example, during the teacher group’s discussion, Jeong questioned the extent to which the Korean government’s social distancing levels reflect the reality of infection:

100 Jeong: Some people don’t visit hospitals even if they have symptoms. The so-called “confirmed cases” are confirmed only when patients already have symptoms, visit a hospital and get diagnosed. That’s how “confirmed cases” statistics are produced. So, even if the confirmed cases were in one digit, there would be a fair chance that the actual cases of infection were much more than that. Tracing patients would be like shutting the stable door after the horse has bolted. So, I disagree with relying on “one-digit confirmed cases” as a condition for school reopening.

Here, Jeong is pointing out that the government statistics cannot fully represent the continuously increasing confirmed cases and thus cannot be used in deciding the conditions for the school reopening. When referring to social distancing levels, the teacher group had a critical discussion about whether the government statistics can actually reflect the reality, instead of simply accepting the statistical evidence.

Comparing how the two groups used the government’s social distancing levels, it can be said that, for the Minister of Education group who focused on reducing criticism from other perspectives, the dependence on the government’s decisions could be useful. This way, the group could rely on the authorities for their decision instead of taking full responsibility for their decision. However, in the teacher group, the PETs were concerned about the responsibilities they would need to bear if virus infections occurred in their classrooms. This concern led them to question the government’s social distancing levels and its use in decision-making. This episode suggests that the reliability of the same evidence (i.e., social distancing levels) was evaluated differently by the Minister group and the teacher group, and thus was used differently in the reasoning process.

## Conclusions and Discussion

### Features of Perspective-Based Socioscientific Decision-Making

Although the presence of multiple perspectives is a core element of socioscientific decision-making (Kahn & Zeidler, 2017), few studies have attended to the value of perspectives in socioscientific reasoning (Covitt et al., 2009; Lee & Grace, 2010). Drawing on the idea of perspective taking in SSI-related decision-making (Kahn & Zeidler, 2019), the current study explored how PETs constructed arguments from different perspectives in the context of COVID-19 and school reopening. Our findings suggest that PETs, when positioned as different stakeholders, sought decisions that could fulfill perspective-specific interests as well as general and common interests such as student safety. The conditions that PETs thought to be sufficient to ensure safety varied and were justified by subjective choices and interpretations of the evidence.

Perspective taking is one of the five components of socioscientific reasoning that Sadler et al. (2007) suggested. Our study shows that, among these components, perspective taking and skepticism can be in conflict in some instances. Specifically, the findings suggest that biased selection and evaluation of evidence could be an unwanted consequence of perspective taking, where emotive reasoning and empathy should be exercised (Newton & Zeidler, 2020). By taking a certain perspective on the issue, the PETs appealed to evidence that supports their initial decision, whereas contradicting evidence was not considered or discussed at length in the group. This points to the need for further efforts to support PETs’ balanced approaches to socioscientific reasoning, where they can both take a specific perspective and be skeptical in the face of new evidence. Such an attitude would be particularly crucial for issues such as COVID-19 since our scientific knowledge about it changes continually based on the available evidence at each moment (Meyerowitz-Katz, 2020). Given that the cherry-picking of evidence has been identified as a characteristic of science denialism (e.g., climate change denial, denial of the health effects of tobacco products) (Conway & Oreskes, 2010), efforts to mitigate the biased use of evidence in decision-making are urgently called for.

Another critical issue in implementing perspective-based socioscientific decision-making activities in the science classroom concerns how to engage learners in such activities meaningfully beyond merely “imagining” what a stakeholder would do based on stereotypes. To support PETs’ perspective-based decision-making, we contextualized the activity within an issue and three perspectives that are relevant to their future roles as elementary teachers and provide justification for their decision. Nevertheless, it is difficult to affirm from the findings to what extent the PETs engaged in the activity from the perspective they took on or simply “predicted” what a certain party would do in a stereotypical way. This is a limitation inherent in many SSI-based activities, and pedagogical strategies will be needed to implement the perspective-based decision-making activities in the science classroom. Considering that the school reopening issue was not a fictional but real-life discussion in society, it could be helpful to ask PETs to compare their own decisions with the actual course of actions taken by the government.

The different types of scientific information (i.e., mechanistic and epidemiological) used for socioscientific decision-making is another interesting finding of the study. Regarding roles of scientific knowledge in SSI-related decision-making, Kolstø (2006) argued that we should shift our focus from whether scientific knowledge is relevant for decision-making to “what kind of knowledge is regarded relevant by different people holding different values” (p. 1710). Mechanistic knowledge was mostly used to emphasize the severity of safety issue in all groups; On the contrary, epidemiological knowledge along with the government’s policy tended to be subjectively chosen and interpreted in support of perspective-dependent claims. To support PETs to use a diverse range of evidence for socioscientific decision-making, it would be helpful to encourage them to engage with both the mechanistic and epidemiological aspects of infectious diseases.

### SSI-Related Decision-Making in Preservice Teacher Education

The ability to make rational decisions on SSIs has become a central quality of responsible citizens in times of global crisis such as COVID-19. Given that teachers are the agents who enact the SSI-related activities in their classrooms, it is crucial for teacher educators to consider how to support science teachers’ understandings of SSIs and SSI-related decision-making (Ladachart & Ladachart, 2021; Ozturk & Yilmaz-Tuzun, 2017; Topcu et al., 2010, 2011). This is particularly so for elementary teachers, who are responsible for developing students’ general abilities through civic, moral, and character education (Benninga, 1991). The features of the PETs’ socioscientific reasoning identified in this study can guide teacher educators on how to support elementary teachers to make responsible decisions that take different perspectival interests into account and thus contribute to resolving social controversies. For instance, hastily arriving at a conclusion despite recognizing the existence of other perspectives narrows down the space for negotiation. Attaching conditions to a claim can be a potential strategy to modify the claim to be less objectionable but might not be an effective strategy to seek mutual agreement with participants in other perspectives.

Considering that PETs will be agents who enact decision-making activities in the classroom as elementary teachers, it would be crucial to support their decision-making practices so that they can enact SSI-related classroom activities themselves. Further research is needed to facilitate the development of PETs’ knowledge and skills in making decisions about SSIs and designing the classroom activities for their students. Although this study was based on the context of school reopening at the time of COVID-19, the implications of the findings can extend to other contemporary SSIs that involve the uncertainty and complexity of scientific knowledge.

## Footnotes

1 Line numbers are not continuous since these conversations were online chats, and for clarity, we omitted line breaks, emojis, and Internet slangs that were unrelated to the decision-making process.
